# Magnetically Driven Living Microrobot Swarms for Aquatic Micro- and Nanoplastic Cleanup

**DOI:** 10.1021/acsnano.5c04045

**Published:** 2025-07-24

**Authors:** Su-Jin Song, Jeonghyo Kim, Roman Gabor, Radek Zboril, Martin Pumera

**Affiliations:** † Advanced Nanorobots & Multiscale Robotics Laboratory, Faculty of Electrical Engineering and Computer Science, VSB − Technical University of Ostrava, 17. listopadu 2172/15, Ostrava-Poruba 70800, Czech Republic; ‡ Nanotechnology Centre, Centre for Energy and Environmental Technologies (CEET), VSB − Technical University of Ostrava, Ostrava-Poruba 70800, Czech Republic; § Regional Centre of Advanced Technologies and Materials, Czech Advanced Technology and Research Institute (CATRIN), Palacky University Olomouc, Olomouc 77146, Czech Republic; ∥ Future Energy and Innovation Laboratory, Central European Institute of Technology, 48274 Brno University of Technology, Purkynova 123, Brno 61200, Czech Republic; ⊥ Department of Medical Research, China Medical University Hospital, China Medical University, No. 91 Hsueh-Shih Road, Taichung 40402, Taiwan

**Keywords:** magnetotactic bacteria, biohybrid microrobots, magnetically driven, swarming behavior, microplastics, nanoplastics, water purification

## Abstract

Micro- and nanoplastic pollution is pervasive worldwide, infiltrating drinking water and food chains, accumulating in the human body, and posing serious threats to public health and ecosystems. Despite these urgent challenges, effective strategies to curb the widespread presence of micro- and nanoplastics have not yet been sufficiently developed. Here, we present magnetically driven living bacterial microrobots that exhibit a nature-inspired three-dimensional (3D) swarming motion, allowing the dynamic capture and retrieval of aquatic micro- and nanoplastics originating from various commercial products. By combining autonomous propulsion with magnetically guided navigation, we enabled the multimodal swarming manipulation of magnetotactic bacteria-based living microrobots (MTB biobots). The actuation of a rotating magnetic field induces a fish schooling-like 3D swarming navigation, allowing the active capture of micro- and nanoplastics, which are then retrieved from the contaminated water by magnetic separation. Our results show that the 3D magnetic swarming of MTB biobots synergistically enhances the removal efficiencies of both model and real-world microplastics, demonstrating their practical potential in water treatment technologies. Overall, plastic-seeking living bacterial microrobots and their swarm manipulation offer a straightforward and environmentally friendly approach to micro- and nanoplastic treatment, providing a biomachinery-based solution to mitigate the pressing microplastic pollution crisis.

## Graphical abstract

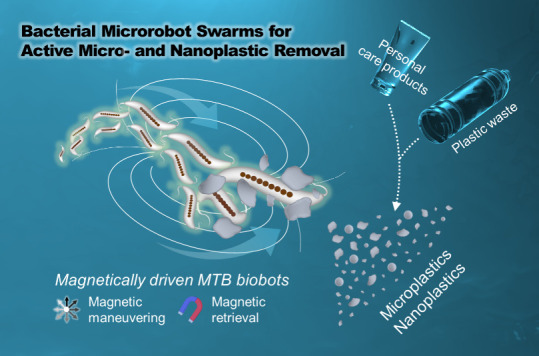

## Introduction

Microplastic and nanoplastic pollution is everywhere, contaminating not only oceans, seafloors, and terrestrial soils but also drinking water and food supplies, posing a significant threat to public health and global ecosystems.^1–4^ In 2019, plastic waste production was estimated at 353 million tons, with 79 million tons of mismanaged plastic waste leaking into the environment through unregulated dumping on land or waterways.^1,5^ With the surging global demand for plastic products, including synthetic textiles, packaging, medical supplies, cosmetics, and personal care products, plastic waste production is predicted to triple, exceeding one billion tons by 2060.^1,3,4^ These are potent sources of microplastics (<5 mm in diameter) and nanoplastics (<1 μm in diameter), which are formed through the breakdown of larger plastic fragments under mechanical wear, heat, UV exposure, and biological processes.^4,6^ These micro- and nanoplastics are difficult to fully degrade under natural conditions, posing a long-lasting ecological threat by infiltrating drinking water and entering all levels of the food chain, eventually leading to human ingestion.^4,6,7^ Numerous studies have reported the presence of microplastics in human tissues and organs, including blood,^8^ liver,^9^ lungs,^10^ placenta,^11,12^ and even breast milk,^13,14^ raising serious concerns about their potential detrimental effects on human health and the risk of translocation to infants. This underscores the urgent need for nanotechnological solutions to effectively collect and remove pervasive micro- and nanoplastics, mitigating unknown critical health risks and environmental impacts.

Micro/nanorobots, small-scale robotic systems capable of precise movement and performing tasks by harnessing surrounding energy sources,^15,16^ are driving advancements across a diverse range of innovative fields, including therapeutics,^17–21^ medical devices,^22,23^ diagnostics,^24–26^ biofilm treatment,^27,28^ water purification,^29–33^ and green energy conversion.^34^ Biohybrid microrobots constructed using engineered bacteria, algae, sperm, and other biological entities are particularly compelling due to their autonomous cellular motility, taxis-based navigation capabilities, and negligible toxicity.^16,35^ Among these, magnetotactic bacteria (MTB) are particularly intriguing as a platform for developing living microrobotic systems. MTB are magnetically responsive microorganisms containing magnetosomes, chain-like organelles of biomineralized magnetite (Fe_3_O_4_) or greigite (Fe_3_S_4_), that enable autonomous propulsion via flagellar motion and alignment along geomagnetic fields to navigate toward optimal environmental conditions.^36–38^ This unique propulsion mechanism, combined with their microaerophilic nature, allows for preferential migration into hypoxic tumor regions, guided and enhanced by external magnetic fields, making them promising candidates for targeted drug delivery in cancer therapy^36,37,39,40^ and biofilm treatment.^41^ In a different context, MTB microrobots also hold great potential for water purification and environmental remediation. The rich functional groups on their cell surfaces can facilitate pollutant bioadsorption through mechanisms such as electrostatic attraction, ion exchange, and complexation.^38,42^ Leveraging their properties, our previous work demonstrated, for the first time, the ability of engineered MTB microrobots to remove organic pollutants from diverse aqueous environments.^42^ The MTB-based biohybrid microrobots offer several distinct advantages for sustainable environmental technologies: (i) MTBs naturally possess adhesive surfaces that can capture pollutants such as heavy metals, organic compounds, and microplastics;^38,42^ (ii) their intrinsic ability to self-propel and respond to magnetic and biochemical stimuli allows for precise wireless maneuverability, enhanced fluid convection, and easy recovery after remediation;^36,37,39–42^ and (iii) as living microorganisms, MTB-based microrobots offer environmentally compatible and biodegradable solutions, minimizing ecological risks, unlike synthetic microrobots that may leave residual materials.^40^ Despite their significant promise, the potential of magnetically responsive bacteria for environmental applications has not yet been sufficiently researched.

Here, we introduce magnetically driven living bacterial microrobots capable of nature-inspired three-dimensional (3D) swarming motion for the dynamic capture and retrieval of aquatic micro- and nanoplastics derived from various commercial products. As outlined in Scheme 1, our approach employs magnetotactic bacteria, strain AMB-1, as living microrobots (MTB biobots). The actuation of MTB biobots combines autonomous self-propulsion by flagellar motion with precise and untethered navigation controlled by diverse magnetic fields, enabling multimodal steering modes such as directional propulsion and rotational circular motion. In particular, actuation within rotating magnetic fields (RMF) in the *X*–*Z* plane induces fish schooling-like 3D swarming of living MTB biobots, enhancing localized fluid convection and facilitating three-dimensional self-navigation. This highly synchronized active motion leads to continuous mechanical collisions that promote physical capture of micro- and nanoplastics, aided by the bacterial surface’s electrostatic, hydrophobic, and biological interactions. Microscopic analyses visually confirmed the dynamic capture and transport of micro- and nanoplastics. Our experiments demonstrated the effective capability of MTB biobots to capture and retrieve both model and real-world micro- and nanoplastics in aqueous environments, with removal efficiencies further enhanced by the collective dynamics of 3D magnetic swarming motion. Overall, plastic-seeking living bacterial microrobots and their swarm manipulation offer a straightforward and environmentally friendly approach to micro- and nanoplastic treatment, providing a biomachinery-based solution to mitigate the pressing microplastic pollution crisis.

**Scheme 1:**
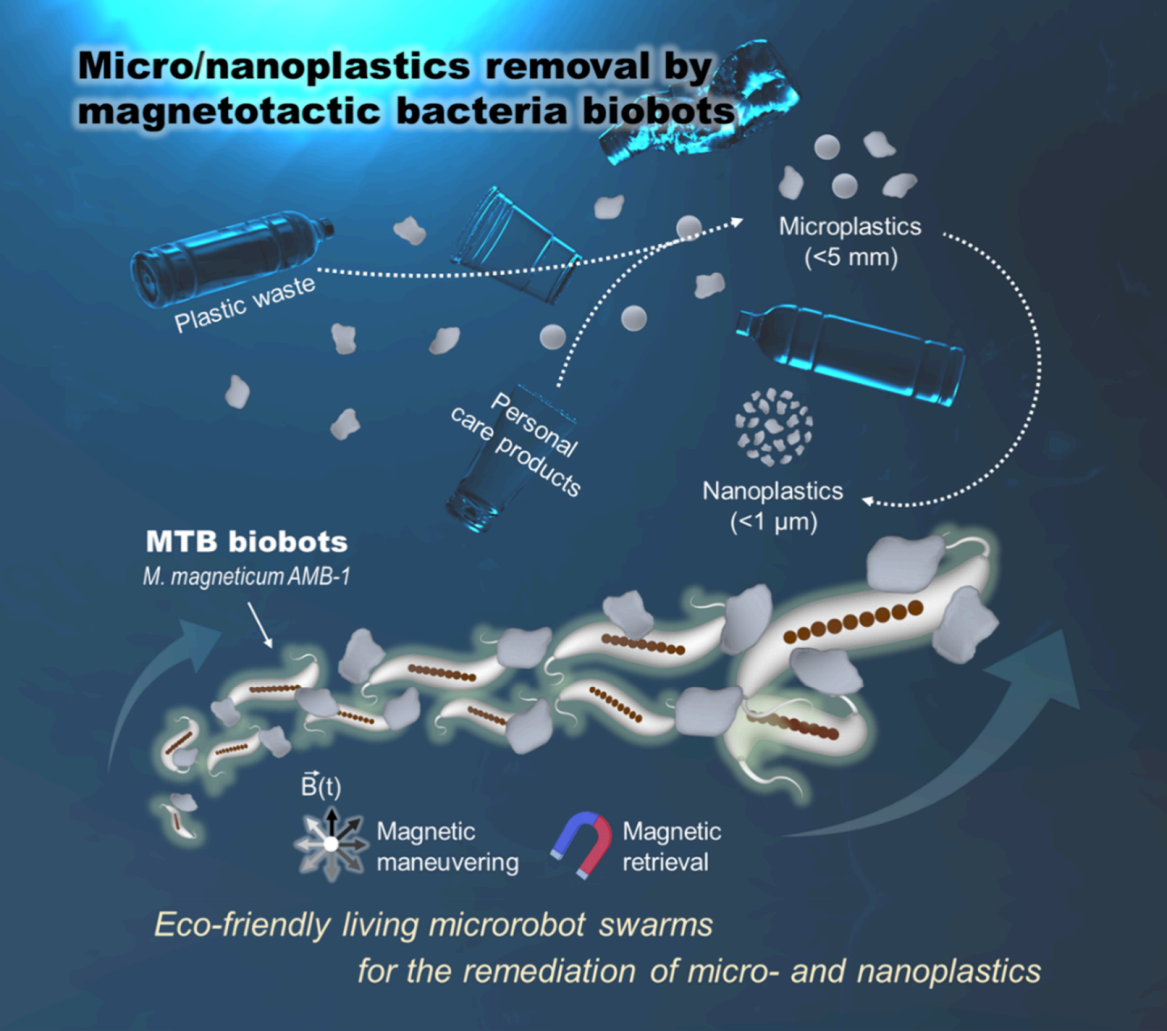
Schematic Illustration of Magnetically Driven MTB Biobot Swarms Designed for the Efficient Removal of Micro- and Nanoplastics from Aquatic Environments^a^

a Under the guidance of programmed rotating magnetic fields, MTB biobots exhibit fish schooling-like 3D swarming behavior to actively capture and retrieve micro- and nanoplastics, including those derived from PET water bottles and body scrubs.

## Results and Discussion

### Preparation and Characterizations of the MTB Biobots

The magnetically responsive bacterium strain AMB-1 was selected as a living biobot for the capture and retrieval of micro- and nanoplastics. Cultured in magnetic spirillum growth medium (MSGM) and standard conditions (see details in the Experimental Section), strain AMB-1 naturally forms intracellular magnetite chains encapsulated within a lipid bilayer, known as magnetosomes.^42–45^ These structures enable the bacteria to align with and respond to external magnetic fields, allowing for magnetically controlled motion.^36,40,42^ The bacterial cultures were grown until the optical density at 565 nm (OD_565_) reached approximately 0.1, leading to the formation of a dark pellet at the bottom of the culture tube, which was subsequently used for further studies (Figure S1, Supporting Information).

The morphological and structural properties of the strain AMB-1 were analyzed using scanning electron microscopy (SEM), transmission electron microscopy (TEM), and energy-dispersive X-ray spectroscopy (EDX). The SEM image reveals that the cultivated strain AMB-1 has a spiral-shaped body with an approximate length of 3 μm and a width of 0.3 μm (Figure 1a). TEM images and EDX mapping further confirm the presence of intracellular magnetosomes with an average diameter of 50 nm, showing carbon (C) distributed throughout the bacterial body, while oxygen (O) and iron (Fe) are arranged in a chain-like nanocrystal structure along the bacterial length (Figure 1d). The X-ray diffraction (XRD) analysis of strain AMB-1 exhibits characteristic 2θ peaks corresponding to magnetite (Fe_3_O_4_), verifying the formation of bacterial magnetosomes (Figure 1b).^46,47^ In addition, the Fourier transform infrared (FTIR) spectrum of strain AMB-1 shows the characteristic peaks of Amide I (1650 cm^–1^) and Amide II (1530 cm^–1^), corresponding to protein absorption. The peaks appeared at ∼1250 and ∼1050 cm^–1^ can be associated with the absorption of lipopolysaccharides (LPS) or phospholipids within the magnetosome membrane.^48,49^ An extra peak observed at 580 cm^–1^ can be attributed to the stretching vibration mode of the Fe–O group (Figure 1c).^50,51^

**Figure 1:**
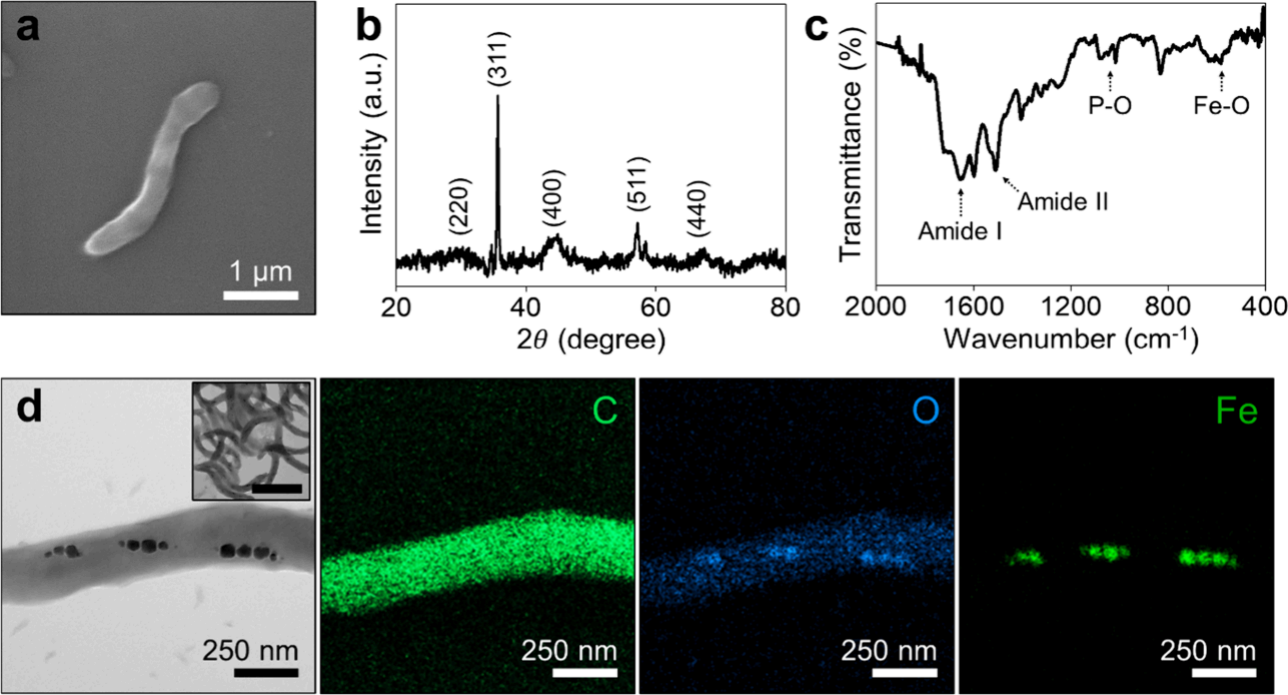
Structural and compositional characterization of MTB biobots. Representative SEM image (a), XRD pattern (b), and FTIR spectrum (c) of strain AMB-1. (d) Different magnification TEM images and corresponding EDX elemental mapping (C, O, and Fe) demonstrating magnetosome chains within strain AMB-1. Inset scale bar: 2 μm.

These results demonstrate that the well-defined structural and compositional properties of strain AMB-1 enable their use as MTB biobots capable of collectively navigating and interacting with micro- and nanoplastics in aqueous environments.

### Magnetically Driven Motion Programming and MTB Biobot Swarms

In their natural environment, MTB, containing anisotropic magnetosomes, exhibit autonomous motility driven by their flagella while aligning along the Earth’s geomagnetic field, presumably facilitating their navigation to growth-favorable zones in aquatic environments.^40,52^ Inspired by their swimming behavior in nature, we aimed to control the collective motions of MTB swarms by applying external magnetic fields (Figure 2). In our experiment, the cultivated strain AMB-1, i.e., the MTB biobots, naturally self-propelled in random directions in the absence of external magnetic fields (Figure 2a,e,i and Supplementary Movie 1). The magnetically driven collective motions of the MTB biobots were controlled using custom-built magnetic field generation systems consisting of two^42^ or three^26,33^ pairs of electromagnetic coils (see the Experiments Section for more details). Additionally, we demonstrated diverse magnetically guided motions by varying the type of magnetic fields. When exposed to a directional magnetic field, the MTB biobots instantly aligned with the applied magnetic fields and propelled in the designated directions, such as rightward, leftward, or along a zigzag trajectory (Figure 2b,f,j and Supplementary Movie 1). Meanwhile, when a rotational magnetic field is applied in the *X*–*Y* plane, the MTB biobots were propelled forward primarily with their flagella, while their magnetic moments dynamically responded to changes in the applied magnetic vectors, resulting in a continuous clockwise circular motion (Figure 2c,g,k and Supplementary Movie 2). Interestingly, applying a rotational magnetic field in the *X*–*Z* plane induces three-dimensional (3D) circular motion, demonstrating collective behavior reminiscent of fish schooling in the ocean (Figure 2d,h,l and Supplementary Movie 3). As demonstrated in Supplementary Movie 3, the direction of the circular rotation plane can be adjusted manually by controlling the angle of the *X*–*Z* rotation plane. Concurrently, the radius of the circular path can be tuned by altering the frequency of the RMF; higher frequencies lead to a gradual reduction in the rotational radius.^45^ The swimming speeds of the MTB biobots under different magnetic-driven modes were calculated based on the representative motions shown in Figures 2e–l and S2, Supporting Information. The autonomous motility of the MTB biobots without magnetic exposure exhibited an average speed of 21.2 ± 2.9 μm s^–1^. Similarly, the average speeds under various magnetic-guided motion modes were measured as 17.2 ± 5.8 μm s^–1^ for directional propulsion, 25.1 ± 2.7 μm s^–1^ for 2D rotational motion, and 21.2 ± 3.3 μm s^–1^ for 3D rotational motion, indicating that magnetic guidance does not affect the forward propulsion speed under the demonstrated experimental conditions (Figure S3, Supporting Information).

**Figure 2:**
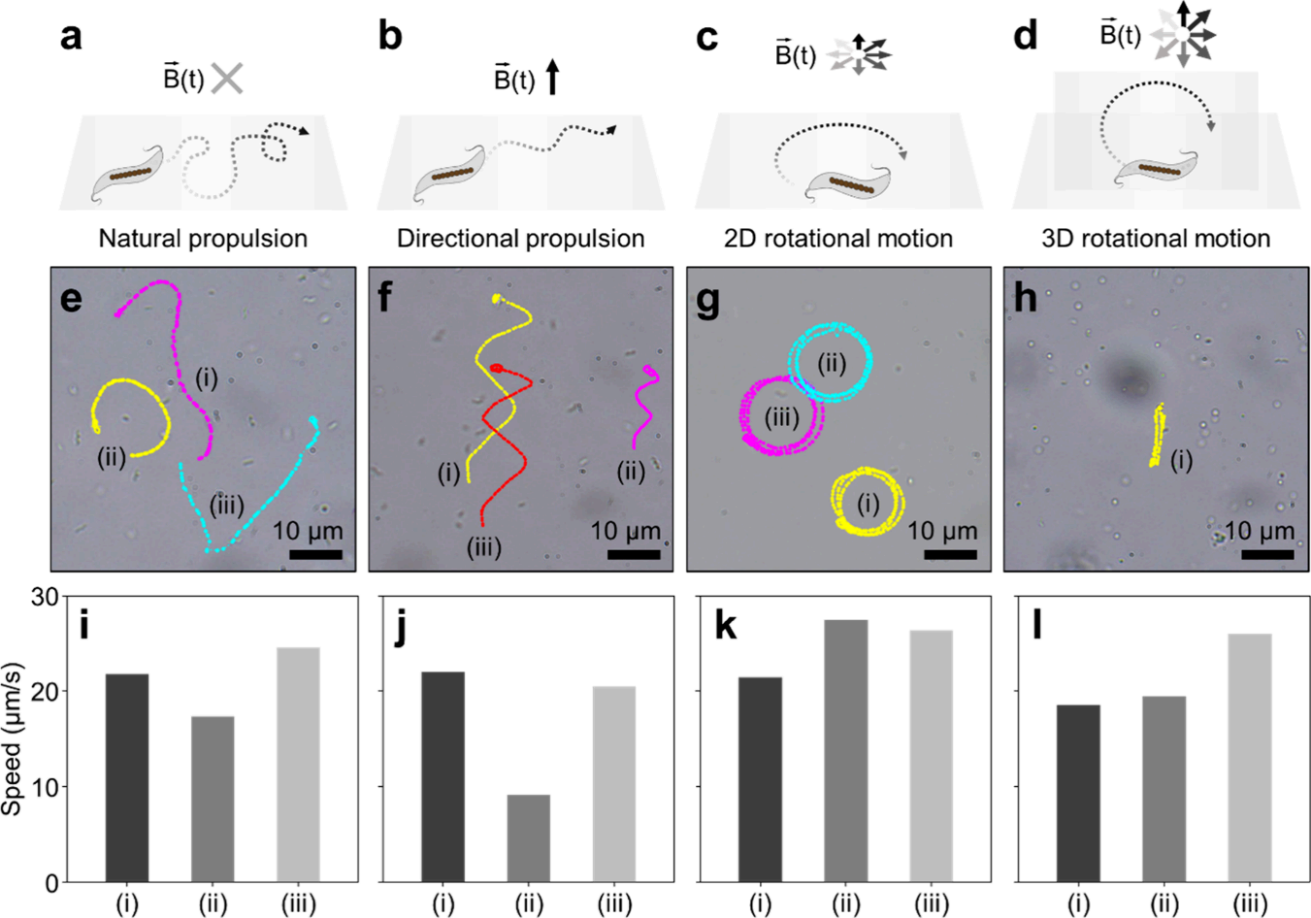
Magnetically driven motion of MTB biobots. (a–l) Conceptual schematics, motion trajectories, speed of MTB biobots under different motion modes: natural propulsion without magnetic guidance (a, e, i) and various magnetically guided motions, including directional propulsion (b, f, j) (see also Supplementary Movie 1), 2D rotational motion in the *X*–*Y* plane (c, g, k) (see also Supplementary Movie 2), and 3D rotational motion in the *X*–*Z* plane (d, h, l) (see also Supplementary Movie 3 and Figure S2, Supporting Information).

### Removal Efficiency of PS Micro- and Nanoplastics: Simulated Model Experiments

The precise and untethered magnetic maneuverability of MTB biobot swarms was demonstrated across multiple steering modes. In particular, the 3D swarming behavior of living MTB biobots provides a unique approach to enhance localized fluid convection and enable three-dimensional self-navigation, offering ecofriendly and sustainable solutions for complex pollution challenges.

By harnessing the nature-inspired 3D magnetic swarming capabilities of living MTB biobots, we proposed an effective and environmentally sustainable strategy for the removal of micro- and nanoplastics in aqueous environments. The experimental procedure is shown schematically in Figure 3a. To evaluate the performance of bacterial microrobotic water purification, we first prepared water samples contaminated with various micro- and nanoplastics. For the initial simulated experiments, fluorescent polystyrene (PS) particles with average diameters of 1 μm and 50 nm were used as a model for micro- and nanoplastics because they have well-defined surface charge properties, a monodisperse size distribution, and easy quantification by fluorescent labeling. The practical applicability was evaluated using microplastics derived from commercial PET bottles and body scrubs, with further details described in the following section. Then, the cultivated MTB biobots, with an optical density of approximately 0.1 at 565 nm (OD_565_), were suspended in contaminated water. Upon activation by RMF (5 mT, 0.5 Hz), the MTB biobots exhibited a synchronized, fish-schooling-like 3D circular swarming motion, enhancing physical interactions and enabling the dynamic capture of micro- and nanoplastics. After a 1 h reaction, the micro- and nanoplastics captured by the MTB biobots were retrieved using an external neodymium magnet (∼1.48 × 10^4^ gauss). The purified water was aspirated from the solution, and its removal efficiency was quantitatively evaluated. Additionally, comprehensive physicochemical analyses were carried out on both the purified water and the retrieved bacterial microrobots.

**Figure 3:**
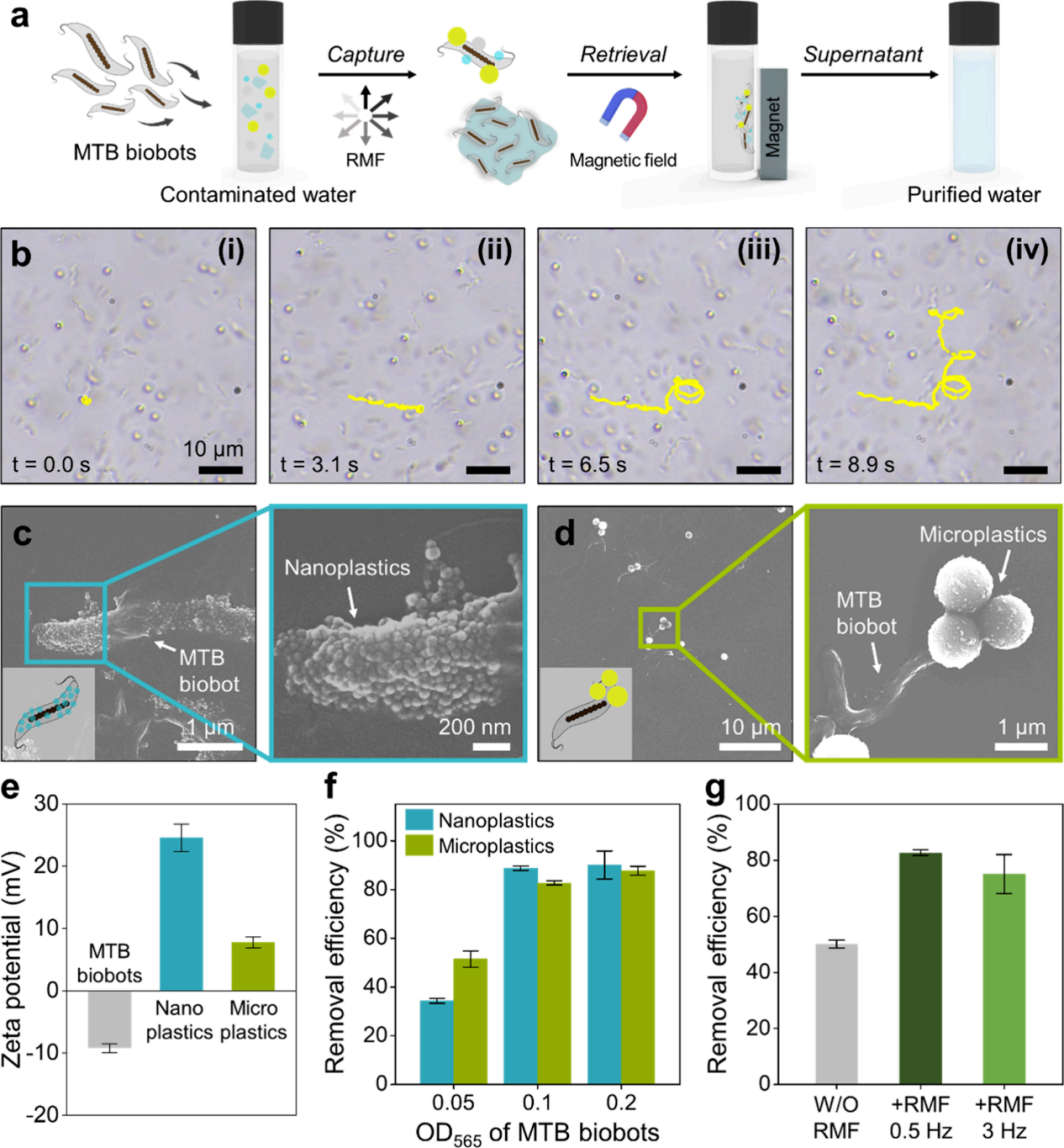
Performance of magnetically driven MTB biobot swarms in the removal of model PS micro- and nanoplastics. (a) Schematic illustrating the experimental procedure for micro- and nanoplastics removal using magnetically driven MTB biobots. (b) Time-lapse images depicting the trajectory of PS microplastic during the dynamic capture process by magnetically driven MTB biobot (see also Supplementary Movie 4). (c, d) SEM images showing the captured PS nanoplastics (c) and PS microplastics (d) after magnetic actuation. (e) Zeta potentials of MTB biobots, PS nanoplastics, and PS microplastics. (f) Removal efficiencies of PS micro- and nanoplastics as a function of MTB biobot concentration. (g) Removal efficiencies of PS microplastics by MTB biobots, with and without RMF actuation. Data in (e), (f), and (g) are presented as mean ± standard deviation from triplicate measurements.

To explore the dynamic capture and transport capabilities of the MTB biobot swarms, a visual microscopic analysis was performed using PS microplastics (Figure 3b and Supplementary Movie 4). Our measurement reveals a weakly negative zeta potential of the MTB biobots (−9.2 ± 0.7 mV), plausibly due to the presence of LPS, phospholipids, and proteins on their cell surface.^53^ To enhance the electrostatic attraction, we used amine-functionalized PS micro- and nanoplastics with positive net surface charge, as shown in Figure 3e. The real-time demonstration is provided in Supplementary Movie 4, with corresponding time-lapse images in Figure 3b. Initially, the MTB biobots were magnetically driven in a clockwise rotational motion, while 1 μm PS microplastics were introduced into the reaction solution (Figure 3b-i). At this stage, the PS microplastics floated along the fluid flow, following a short linear trajectory (Figure 3b-ii). Within seconds, the navigating MTB biobot swarms captured and transported the PS microplastics, as indicated by a shift in the microplastics’ trajectory to a circular motion (Figure 3b-iii,iv). This result demonstrates that the MTB biobot swarms can instantaneously capture and efficiently remove microplastics.

The low- and high-magnification SEM images of the recovered MTB biobots after a 1 h removal reaction with PS micro- and nanoplastics are shown in Figure 3c,d. Due to their relatively small size (50 nm), PS nanoplastics are abundantly adsorbed on the cell surface of MTB biobots (Figure 3c). Conversely, PS microplastics, which are comparable in size to MTB biobots, tend to adsorb in clusters of two or three plastics (Figure 3d). These observations further confirm that PS micro- and nanoplastics are effectively captured during the reaction and adhere strongly, although there are size-dependent differences in their adsorption behavior.

After the effective binding capability was confirmed, the removal efficiencies of MTB biobots were quantitatively evaluated to examine their capability in eliminating PS micro- and nanoplastics. Using fluorescently labeled PS micro- and nanoplastics, we quantified concentrations before and after treatment with MTB biobots by measuring their strong characteristic peaks at 500 nm for PS microplastics and 420 nm for PS nanoplastics (Figure S4, Supporting Information). The removal efficiency of micro- and nanoplastics was calculated using eq 1):

$$\mathrm{Removal}\;\mathrm{Efficiency}\;(\%)=\frac{C_{i}-C_{t}}{C_{i}}\times 100$$ 1

where *C*_*i*_ is the initial concentration of micro- and nanoplastics in the tested solution, and *C*_*t*_ is the final concentration after the reaction. Upon 3D magnetic swarming actuation, MTB biobots significantly reduced the fluorescence signals of PS micro- and nanoplastics through active capture and retrieval. As shown in Figure 3f, removal efficiencies increase with MTB biobot concentration, rising from an OD_565_ of 0.05 to 0.1 and reaching saturation at higher concentrations for both PS micro- and nanoplastics. To further investigate the effect of 3D magnetic swarming motion, Figure 3g compares the removal efficiencies of PS microplastics by MTB biobots with and without RMF actuation. In the absence of magnetic actuation, MTB biobots relying solely on natural bacterial propulsion achieved a removal efficiency of 50%. In contrast, magnetically actuated MTB biobots exhibited enhanced performance, with removal efficiencies of 83% at 0.5 Hz and 75% at 3 Hz, representing increases of 33 and 25%, respectively. The greater efficiency observed at 0.5 Hz compared to at 3 Hz is plausibly due to the generated motion with larger circular trajectories under lower-frequency RMF, which could improve the possibility of capturing dispersed microplastics, as illustrated in Supplementary Movie 3. Figure S5, Supporting Information, which presents the removal performance of PS nanoplastics with and without RMF actuation. Although magnetically actuated 3D swarming motion improved removal efficiencies compared to natural bacterial propulsion, reaching 89% at 0.5 Hz and 87% at 3 Hz, the enhancement was relatively modest (∼4–6%). This limited improvement, consistent with previous reports,^30^ is likely attributed to the ultrasmall size of the PS nanoplastics (50 nm) and their engineered strong electrostatic affinity for the bacterial surface, making them readily captured even under natural bacterial propulsion, as evidenced by SEM images (Figure 3c) and zeta potential measurements (Figure 3e). Based on these results, an OD_565_ of 0.1 and RMF actuation at 5 mT and 0.5 Hz were selected as the optimized conditions for subsequent experiments.

We further examined the removal capability of MTB biobots in various aqueous media, including bottled drinking water, tap water, and river water. Previous studies have demonstrated the robust motility of MTB biobots in such complex water matrices, which is a key feature for effective contaminant binding and removal.^42^ As illustrated in Figure S6, Supporting Information, the MTB biobots exhibit effective removal of PS microplastics in all tested media: 80% in bottled drinking water, 79% in tap water, and 77% in river water, comparable to their performance in distilled water (83%). These results indicate minimal interference from realistic aqueous environments, offering strong potential for magnetically actuated MTB biobots for micro- and nanoplastic remediation in contaminated water systems.

### Cleaning Microplastics Derived from PET Bottles and Body Scrubs: Toward Real-World Aquatic Micro- and Nanoplastics Treatment

Beyond simulated model experiments with PS micro- and nanoplastics, the practical applicability of living bacterial microrobots was investigated using microplastics derived from commercially available PET bottles and body scrubs. PET microplastics were produced by grinding the surface of postconsumer PET water bottles and collecting the resulting fine fragments. Microplastics from body scrubs were extracted from commercial cleansing products and rinsed thoroughly before use in subsequent experiments.

The experiments to test PET and body scrub microplastics followed the same procedure established for PS micro- and nanoplastics. The MTB biobots were introduced into contaminated water samples containing PET and body scrub microplastics and activated into a 3D magnetic swarming motion by RMF. The effective capture and retrieval of PET and body scrub microplastics were confirmed by optical microscopy (Figure 4a,b) and SEM (Figure 4c,d) analyses. PET and body scrub microplastics were predominantly recovered as aggregates with the MTB biobots, while microscopy images of the supernatant solution showed no remaining microplastics or biobots, indicating successful removal of microplastics (Figure 4a,b).

**Figure 4:**
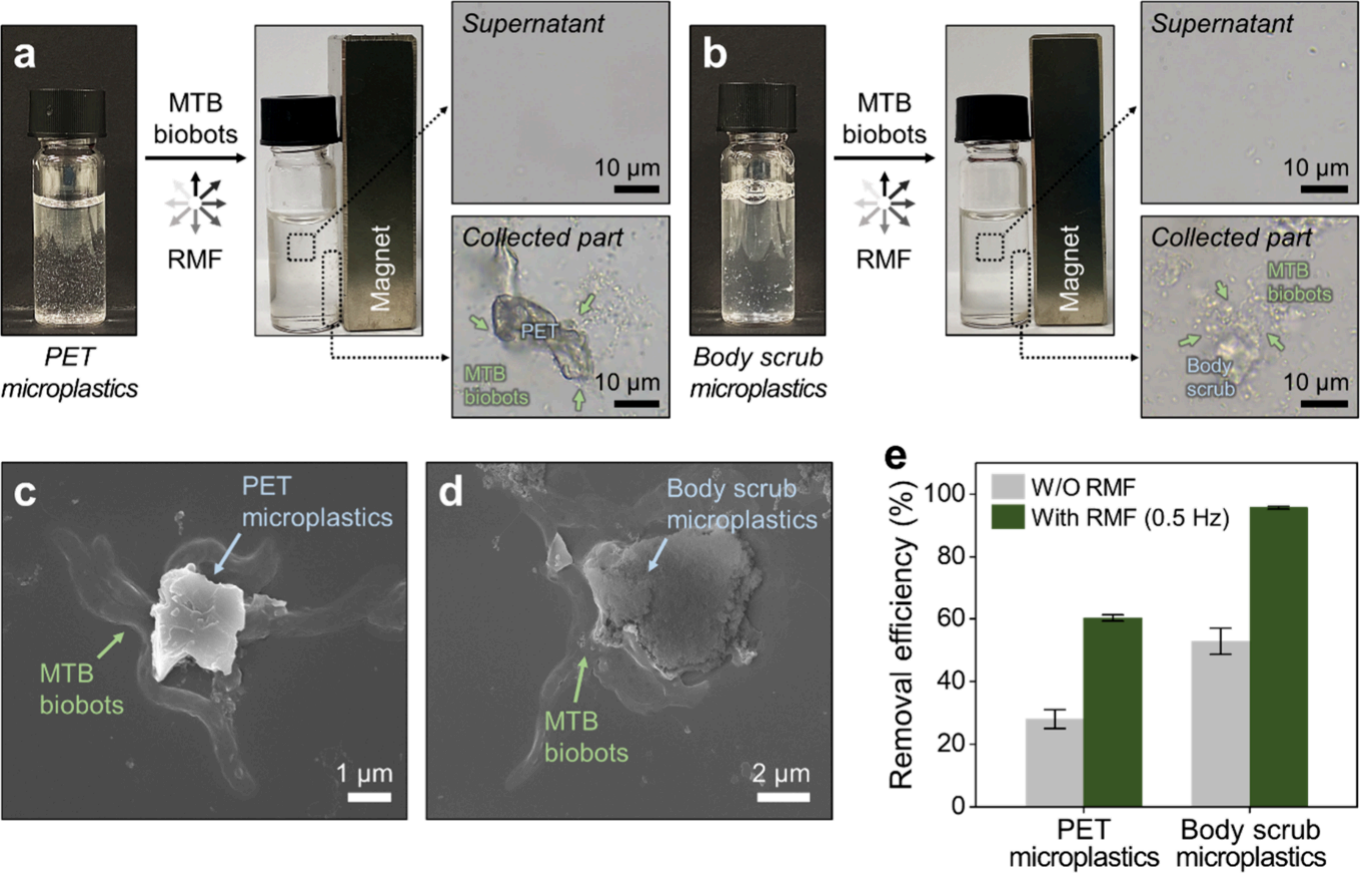
Magnetically driven MTB biobot swarms for the removal of microplastics derived from commercially available PET bottles and body scrubs. Removal of PET (a) and body scrub (b) microplastics by magnetically driven MTB biobots, with optical microscopy images of the supernatant and magnetically collected samples. (c, d) SEM images showing PET (c) and body scrub (d) microplastics captured by MTB biobots. (e) Removal efficiencies of PET and body scrub microplastics by MTB biobots, with and without RMF actuation. In the optical microscopy (a, b) and SEM (c, d) images, green arrows denote MTB biobots, while blue arrows indicate microplastics. Data in (e) are presented as mean ± standard deviation from triplicate measurements.

Our results also demonstrate the effective adhesion and coaggregation of MTB biobots with microplastics. The actively moving MTB biobots, exhibiting synchronized circular motion in three-dimensional space, continuously collide with microplastic surfaces, which often feature irregularities, rough textures, and crevices that can promote nonspecific physical adhesion to microplastics.^7,54,55^ Additionally, the bacterial cell surface and associated organelles, containing polysaccharides, phospholipids, and proteins, help to reduce repulsive forces and contribute to electrostatic, hydrophobic, and biological attraction to microplastics (Figure 1c).^56–58^ Overall, the enhanced mechanical and physicochemical interactions driven by bacterial microrobotic motility facilitate physical bioadsorption onto microplastics of varying sizes and compositions, enabling their capture and retrieval.

To quantify the removal efficiency, PET and body scrub microplastics were stained with Nile Red. Nile Red is a hydrophobic fluorescent dye that is widely used for the effective staining of various micro- and nanoplastics, such as polystyrene, polypropylene, and polyethylene, due to its strong hydrophobic affinity to plastic surfaces.^59,60^ The Nile Red-stained PET and body scrub microplastics exhibited strong emission at ∼630 nm, which was monitored to determine the removal efficiency (Figure S7, Supporting Information). Under the 3D magnetic swarming driven by RMF (5 mT, 0.5 Hz), the MTB biobots led to a substantial decrease in fluorescence signals, achieving removal efficiencies of 60% for PET microplastics and 96% for body scrub microplastics within a 1 h reaction (Figure 4e). By contrast, control experiments performed without magnetic actuation resulted in significantly lower removal efficiencies, with 28% for PET microplastics and 53% for body scrub microplastics, indicating a decrease of 32–43%, respectively. The observed differences in removal performance between PET and body scrub microplastics are attributed to the distinct physicochemical characteristics of the microplastics. PET microplastics, characterized by higher rigidity and a negative surface charge, displayed reduced bacterial adhesion, whereas body scrub microplastics, possessing softer and neutral surfaces, interacted more readily with MTB biobots.^56^ These results highlight the essential role of magnetically driven 3D swarming in enhancing the capture and removal of microplastics derived from commercial products.

Our study also demonstrated real-time microscopic observation of PET and body scrub microplastics retrieval to further investigate their strong adhesion and versatile isolation capabilities. Figure 5a depicts the process by which magnetically driven MTB biobot swarms adhere to and retrieve PET and body scrub microplastics, forming aggregates even with microplastics larger than the bacterial cells. Microscopy images show multiple MTB biobots tightly bound to the surfaces of these microplastics following magnetic actuation (Figure 5b,c). The MTB biobot-microplastic aggregates were then retrieved from the solution by using an external neodymium magnet (∼1.32 × 10^4^ gauss). Supplementary Movie 5 illustrates the transport of ∼10 μm-sized PET and body scrub microplastics in the direction of the applied static magnetic field. Despite the relatively large size and weight of the microplastics, the coordinated agglomeration of multiple MTB biobots enables effective transport under magnetic guidance (Figure 5d,e). Under these conditions, transport speeds of captured microplastics were measured at 4.9 μm s^–1^ for PET microplastic aggregates and 2.2 μm s^–1^ for body scrub microplastic aggregates. We further demonstrated the magnetic retrieval of PET microplastics captured by MTB biobots in a test vial using an external magnetic field, revealing strong adhesion and robust magnetic responsiveness observable at the macroscopic level, as shown in Supplementary Movie 6. These results underscore the potential of MTB biobot swarms for the straightforward retrieval of microplastics originating from various commercial products, thereby reducing their release into ecological environments.

**Figure 5:**
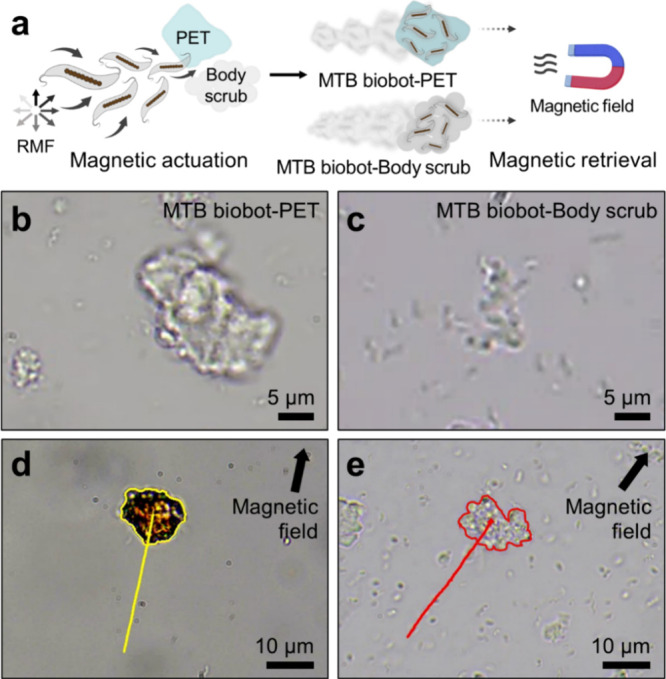
Efficient magnetic retrieval of PET and body scrub microplastics using MTB biobots. (a) Schematic illustrating the dynamic adsorption and magnetic retrieval process of PET and body scrub microplastics by MTB biobot swarms. (b, c) Optical microscopy images showing PET (b) and body scrub (c) microplastics captured by MTB biobots. (d, e) Microscopic demonstration of the magnetic retrieval of PET (d) and body scrub (e) microplastics, guided by an external magnetic field (see also Supplementary Movie 5). Average retrieval speeds were measured at 4.9 μm s^–1^ for PET and 2.2 μm s^–1^ for body scrub microplastics.

### Biocompatibility and Environmental Safety of MTB Biobots

The use of living bacterial microrobots for micro- and nanoplastic remediation offers a novel, nature-inspired strategy that harnesses the inherent capabilities of magnetotactic microorganisms. However, introducing such biohybrid systems into aquatic environments requires a thorough evaluation of their ecological safety. To elucidate their environmental compatibility, we examined the biocompatibility of MTB biobots and evaluated their potential to release harmful substances or secondary contaminants into water systems.

MTB are generally considered as nontoxic and biocompatible, with previous studies demonstrating minimal cytotoxic effects within defined concentration ranges in both in vitro and in vivo models.^40,61^ These characteristics have driven increasing interest in their biomedical applications, particularly in targeted drug delivery and precision therapeutics.^36,37,39,40^ Given the importance of low cytotoxicity for environmental applications, we examined the cytotoxicity of MTB biobots ( strain AMB-1) using HT1080 human fibrosarcoma cells (Figure S8a, Supporting Information). Cells were incubated for 24 h with varying concentrations of MTB biobots (0.4–100% v/v; OD_5__6__5_ = 0.1 defined as 100%), and cell viability was quantified using the MTS assay. Across all tested concentrations, no significant reduction in cell viability was observed, indicating no acute toxicity at levels used for micro- and nanoplastic remediation.

To investigate the potential release of harmful bacterial byproducts, such as endotoxins, during the magnetic actuation of MTB biobots in aqueous media, we further evaluated the environmental compatibility of the reacted supernatant solution. After 1 h of actuation under an RMF (5 mT, 0.5 Hz) and complete retrieval of the MTB biobots (OD_565_ = 0.1), supernatants were collected and subjected to cytotoxicity and endotoxin quantification assays. The cytotoxicity test performed after 24 h exposure to HT1080 human fibrosarcoma cells showed no significant decrease in cell viability across all tested concentrations (Figure S8b, Supporting Information). In parallel, endotoxin levels were quantified using a chromogenic Limulus amebocyte lysate (LAL) assay, revealing negligible endotoxin concentrations (0.085 ± 0.007 EU mL^–1^), which are comparable to those found in distilled water (0.048 ± 0.021 EU mL^–1^) and substantially below the Food and Drug Administration (FDA)-recommended safety threshold of 0.5 EU mL^–1^ for injectable products (Figure S9, Supporting Information).^62^ These results confirm that magnetic actuation of the MTB biobot under the tested conditions poses minimal cytotoxicity and endotoxin-related risks, supporting their environmental compatibility and applicability in aqueous systems.

## Conclusions

In this study, we proposed magnetically driven swarms of living bacterial microrobots for the cleanup of aquatic micro- and nanoplastics. The MTB biobots, derived from the strain AMB-1, exhibit intrinsic magnetic responsiveness and adhesive cell surfaces, making them fascinating platforms for living microrobotic systems. By combining autonomous propulsion with magnetically guided navigation, we enabled multimodal swarming manipulation in both 2D and 3D geometries. Particularly, the MTB biobots demonstrated fish schooling-like 3D swarming motion under RMF, enhancing fluid convection and facilitating self-navigation toward micro- and nanoplastics in 3D spaces. This synchronized 3D swarming navigation improved mechanical and physicochemical interactions, promoting the effective capture and coaggregation of micro- and nanoplastics. The dynamic capture and retrieval of micro- and nanoplastics were visually confirmed by real-time optical microscopy analyses. The MTB biobots achieved removal efficiencies of 83–89% for model PS micro- and nanoplastics, and 60–96% for microplastics derived from PET water bottles and body scrubs. The actuation of 3D magnetic swarming motion led to a 6–43% improvement in removal performance, with more pronounced effects observed for larger microplastics and those with relatively weak electrostatic interactions with the bacterial surface. Our results demonstrate that the magnetically driven MTB biobots are capable of effectively capturing and retrieving both model and real-world microplastics, offering a straightforward and environmentally friendly approach for water purification. The promise of living bacterial microrobots and their swarm behavior provides a biomachinery-based solution to mitigate the pressing microplastic pollution crisis.

## Materials and Methods

### *M. magneticum* Strain AMB-1 Culture

 strain AMB-1 (ATCC 700264) was cultured in revised magnetic spirillum growth medium (MSGM; ATCC, Medium 1653), which contained 5 mL of Wolf’s Mineral solution, 0.68 g of potassium phosphate (KH_2_PO_4_), 0.37 g of succinic acid, 0.37 g of tartaric acid, 0.12 g of sodium nitrate (NaNO_3_), 0.035 g of ascorbic acid, and 0.05 g of sodium acetate per 1 L of distilled water.^42,44^ The final pH of MSGM was adjusted to 6.75 with 5 M NaOH before autoclaving. Before culture, 10 mL of Wolf’s Vitamin Solution and 2 mL of 0.1 M ferric quinate were supplemented to the autoclaved MSGM. The strain AMB-1 was grown under anaerobic conditions at 30 °C for 7 days.

### Characterization of MTB Biobots

The morphology and magnetosome crystals of the strain AMB-1 were characterized using scanning electron microscopy (SEM, MAIA3, Tescan, Czech Republic), transmission electron microscopy (TEM, JEOL JEM-2200FS, Japan), and energy-dispersive X-ray spectroscopy (EDX, Oxford Instruments, UK). strain AMB-1 obtained by centrifugation (8000 rpm, 15 min) was fixed in 5% glutaraldehyde dissolved in 0.1 M phosphate-buffered saline (pH 7.2) at room temperature for 1 h. Afterward, the cells were washed with distilled water and dehydrated through an ethanol series ranging from 40 to 100%. To obtain SEM images of MTB biobots after microplastic capture, the samples were collected by using a neodymium magnet, redispersed in distilled water, and completely dried at 30 °C. X-ray diffraction (XRD) analysis was carried out using an X-ray diffractometer (Bruker, D8-Advance, Germany). The Fourier-transform infrared (FTIR) spectrum was measured by a Nicolet 6700 FTIR spectrometer (Thermo-Nicolet, USA). Surface potentials were measured using a zeta-sizer (Zetasizer Pro, Malvern Instruments Ltd., UK).

### Magnetic-Driven Motion Study

 strain AMB-1 can be controlled by an external magnetic field due to its intrinsic magnetic properties, as detailed in a previous study.^42^ Briefly, the magnetic-driven motion studies were carried out using custom-built magnetic field generation systems consisting of two^42^ or three^26,33^ pairs of electromagnetic coils mounted on a 3D-printed coil holder. Directional propulsion navigation is achieved via an externally connected joystick, allowing the MTB biobots to move along the directional magnetic field. 2D and 3D rotational motions are controlled by RMF. The motion of MTB biobots and their interactions with micro- and nanoplastics were investigated using an inverted optical microscope (Olympus IX73) equipped with a Basler acA-1920-155 μm monochrome CMOS camera and a 50× objective lens. Recorded videos were analyzed by using NIS-Elements AR 3.2 software to determine average velocities and tracking trajectories.

### Preparation of Micro- and Nanoplastics

The simulated model experiments were performed with commercial PS microparticles (1.0 μm, amine-modified fluorescent labeled polystyrene, L1030, Sigma-Aldrich) and nanoparticles (0.05 μm, amine-modified fluorescent labeled polystyrene, L0780, Sigma-Aldrich). PS micro- and nanoplastic stock solutions were diluted 20:1 in distilled water and then used for further quantification experiments. The retrieval experiments targeting PET and body scrub microplastics were prepared from commercially available products. PET microplastics were produced by grinding the surface of postconsumer PET water bottles and collecting the resulting fine fragments. Microplastics from body scrubs were extracted from commercial cleansing products and rinsed thoroughly before use in the subsequent experiments. Before use, both plastic samples were finely ground using a homogenizer, redispersed in DW (1 mg/mL), and then used for experiments.

### Microplastic Capture and Retrieve

The removal efficiency of micro- and nanoplastics by MTB biobots was carried out in four different samples: PS micro- and nanoplastics, and PET and body scrub microplastics. MTB biobots resuspended in DW at a concentration of OD_565_ = 0.1 were used. The microrobotic capture was conducted using a magnetic manipulation system with an *X*–*Z* plane RMF in random angle change mode at 5 mT and 0.5 Hz. After the reaction, to evaluate the removal efficiency of micro- and nanoplastics, a permanent neodymium magnet was used to collect the MTB biobots (6 × 0.5 × 1.5 cm, 1.48 × 10^4^ gauss). The experimental vials were placed on a neodymium magnet, allowing MTB biobot–microplastic aggregates and unbound MTB biobots to be magnetically retrieved over a 30 min period. The resulting purified supernatant was then collected for further analysis. The removal efficiencies were quantified using a spectrofluorometer (Jasco FP-8550) and visualized using SEM and optical microscopy. The removal of PET and body scrub microplastics by MTB biobots was quantified by staining with Nile Red. Ten mg of PET and body scrub microplastics were mixed with a 10 mM Nile Red solution and shaken overnight, then washed with ethanol, and stored for quantification. All experiments were repeated three times.

## Supplementary Material















## Notes

Detailed methods for the cytotoxicity test and endotoxin quantification assay; optical density measurements to evaluate the growth of strain AMB-1; trajectories of 3D rotational motion; comparison of MTB biobot speeds; fluorescence spectra of PS micro- and nanoplastics; removal efficiencies of PS nanoplastics with and without RMF actuation; removal efficiencies of PS microplastics in various aqueous environments; fluorescence spectra of nile red-stained PET and body scrub microplastics; cytotoxicity evaluation; and endotoxin quantification assay (PDF)
Natural propulsion and magnetic directional guidance of MTB biobots (MP4)
2D rotational motion of MTB biobots in the *X*–*Y* plane (MP4)
3D rotational motion of MTB biobots in the *X*–*Z* plane (MP4)
Magnetically driven MTB biobots capturing PS microplastics (MP4)
Efficient magnetic retrieval of PET and body scrub microplastics using MTB biobots (MP4)
Magnetic retrieval of PET microplastics captured by MTB biobots in a vial (MP4)
