# Supplementary material for: Magnetically Driven Living Microrobot Swarms for Aquatic Micro- and Nanoplastic Cleanup
Source: ACS Nano. 2025 Jul 24;19(30):27259–69. doi: 10.1021/acsnano.5c04045 (PMC12333422; doi:10.1021/acsnano.5c04045)
Supplement: Supplementary file 1 [file nn5c04045_si_001.pdf]

Supporting Information

Magnetically Driven Living Microrobot Swarms for Aquatic Micro- and Nanoplastic Cleanup

*Su-Jin Song[1], Jeonghyo Kim[1], Roman Gabor[2], Radek Zboril[2,3], Martin Pumera[1,4,5]**

[1]Advanced Nanorobots & Multiscale Robotics Laboratory, Faculty of Electrical Engineering and Computer Science, VSB – Technical University of Ostrava, Ostrava-Poruba 70800, Czech Republic

[2]Nanotechnology Centre, Centre for Energy and Environmental Technologies (CEET), VSB – Technical University of Ostrava, Ostrava-Poruba 70800, Czech Republic

[3]Regional Centre of Advanced Technologies and Materials, Czech Advanced Technology and Research Institute (CATRIN), Palacky University Olomouc, Olomouc 77146, Czech Republic

[4]Future Energy and Innovation Laboratory, Central European Institute of Technology, Brno University of Technology, Brno 61200, Czech Republic

[5]Department of Medical Research, China Medical University Hospital, China Medical University, Taichung 40402, Taiwan

*Corresponding author: pumera.research@gmail.com

TABLE OF CONTENTS

1. Supplementary Methods
2. **Figure S1.** Optical density measurements at 565 nm (OD_{565}) to evaluate the growth of *M. magneticum* strain AMB-1.
3. **Figure S2.** Additional trajectories illustrating the 3D rotational motion of MTB biobots corresponding to Figure 2l.
4. **Figure S3.** Comparison of the speed of MTB biobots under different motion modes.
5. **Figure S4.** Fluorescence spectra of PS nanoplastics (a) and PS microplastics (b) during the 1-hour removal reaction by the MTB biobot swarm (OD_{565} = 0.1), with and without magnetic actuation (5 mT, 0.5 Hz).
6. **Figure S5.** Removal efficiencies of PS nanoplastics by MTB biobots (OD_{565} = 0.1) with and without RMF actuation.
7. **Figure S6.** (a) Removal efficiencies of PS microplastics by MTB biobots (OD_{565} = 0.1) actuated under a RMF (5 mT, 0.5 Hz) in various aqueous environments, including distilled water, drinking water, tap water, and river water. (b) Geographical location of the river water sampling site (the Ostravice River, Ostrava, Czech Republic).
8. **Figure S7.** Fluorescence spectra of Nile Red-stained PET microplastics (a) and Nile Red-stained body scrub microplastics (b) before and after removal treatment using magnetically driven MTB biobots.
9. **Figure S8.** Cytotoxicity evaluation of MTB biobots and supernatant solution after MTB biobot operation.
10. **Figure S9.** Quantification of endotoxins in the supernatant following MTB biobot operation.
11. **Supplementary Movie 1.** Natural propulsion and magnetic directional guidance of MTB biobots.
12. **Supplementary Movie 2.** 2D rotational motion of MTB biobots in the X-Y plane.
13. **Supplementary Movie 3.** 3D rotational motion of MTB biobots in the X-Z plane.
14. **Supplementary Movie 4.** Magnetically driven MTB biobots capturing PS microplastics.
15. **Supplementary Movie 5.** Efficient magnetic retrieval of PET and body scrub microplastics using MTB biobots.
16. **Supplementary Movie 6.** Magnetic retrieval of PET microplastics captured by MTB biobots in a vial

Supplementary Methods

Cytotoxicity tests

The MTS assay was carried out to determine the cytotoxicity effects of MTB biobots and the supernatant collected after magnetic actuation and retrieval. MTB biobots were first prepared at an OD_{565} of 0.1 and tested to evaluate their direct cytotoxicity. Subsequently, supernatant solutions were collected following 1 hour of RMF actuation (5 mT, 0.5 Hz) and complete retrieval of the MTB biobots at the same concentration (OD_{565} = 0.1). These two samples were examined to assess both the intrinsic bacterial toxicity and the potential release of harmful substances into the aqueous environment. HT1080 cells were seeded at a density of 5,000 cells per well in 90 µL of HEPES-buffered Minimum Essential Medium (MEM) supplemented with 10% fetal bovine serum, 0.1 mM non-essential amino acids, 1 mM sodium pyruvate, and 2 mM L-glutamine in 96-well plates. Samples were prepared by diluting stock solutions of each sample in deionized water to achieve final concentrations ranging from 100% to 0.4% (v/v). Then, 10 µL of each diluted sample was added to the wells to reach a final volume of 100 µL (n=3). Plates were incubated at 37 °C for 24 hours. For supernatant samples, incubation was carried out in a 5% CO_2 incubator, while bacterial samples were incubated in a non-CO_2 incubator. In both cases, HEPES-buffered media ensured pH stability in the absence of CO_2. Following incubation, the media was aspirated and replaced with 100 µL of fresh complete media. MTS reagent (20 µL per well) was then added, and plates were incubated for 4 hours at 37 °C. Absorbance was measured at 490 nm using a microplate reader.

Endotoxin quantification assay

To evaluate potential endotoxin release following MTB biobot exposure and their magnetic actuation in aqueous media, supernatant samples were collected after 1 hour of RMF actuation (5 mT, 0.5 Hz) and complete retrieval of the MTB biobots at $OD_{565} = 0.1$. Endotoxin levels in the supernatant were quantified using the Pierce™ Chromogenic Endotoxin Quant Kit (Thermo Fisher Scientific, USA), following the manufacturer's instructions. Distilled water was used as a negative control for comparison. Briefly, 50 µL of the sample was added into a 96-well microplate, followed by the addition of 50 µL of LAL reagent. The plate was incubated at 37°C for 14 minutes, and then 100 µL of chromogenic substrate was added. After an additional 6-minute incubation, the reaction was stopped by adding 100 µL of stop solution, and absorbance was measured at 405 nm using a TECAN Infinite M Plex microplate reader (n = 3).

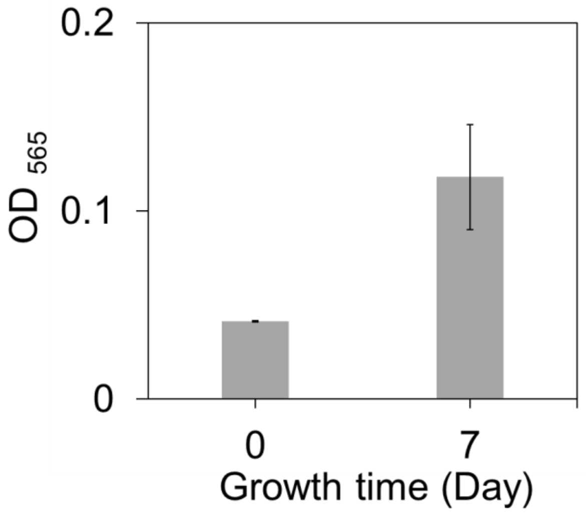

Figure S1. Optical density measurements at 565 nm (OD_{565}) to evaluate the growth of *M. magneticum* strain AMB-1. Data are presented as mean ± s.d. from triplicate measurements.

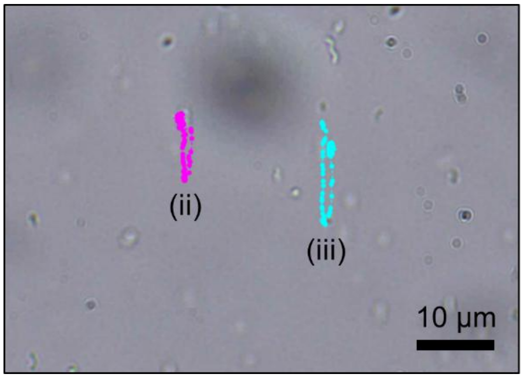

Figure S2. Additional trajectories illustrating the 3D rotational motion of MTB biobots corresponding to Figure 2l.

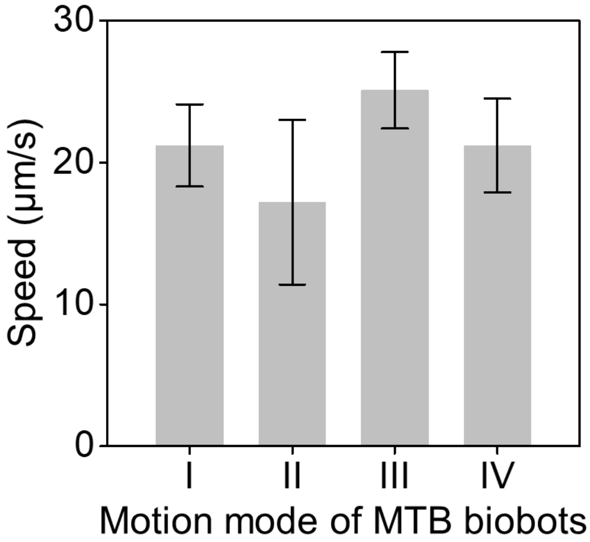

Figure S3. Comparison of the speed of MTB biobots under different motion modes: (I) natural bacterial propulsion, and various magnetically guided motions, including (II) directional propulsion, (III) 2D rotational motion in the X–Y plane, and (IV) 3D rotational motion in the X–Z plane. Data are presented as mean ± s.d. from triplicate measurements.

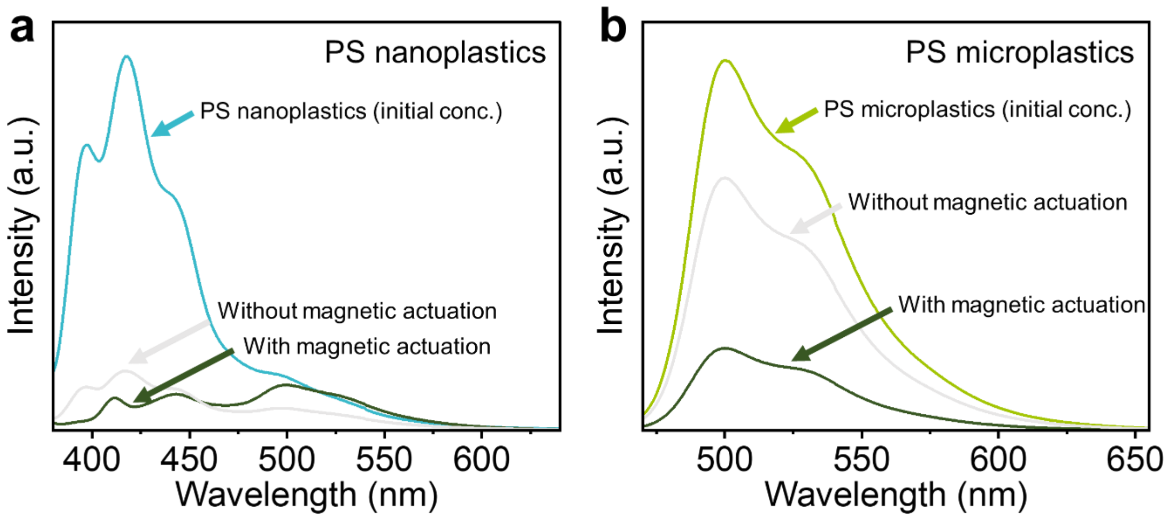

Figure S4. Fluorescence spectra of PS nanoplastics (a) and PS microplastics (b) during the 1-hour removal reaction by the MTB biobot swarm (OD_{565} = 0.1), with and without magnetic actuation (5 mT, 0.5 Hz).

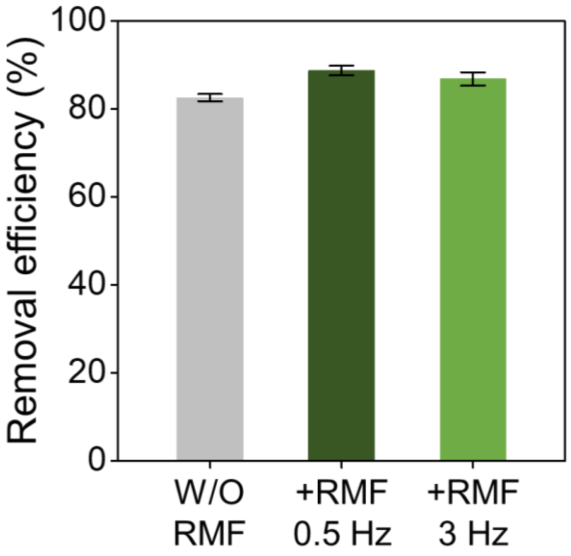

Figure S5. Removal efficiencies of PS nanoplastics by MTB biobots (OD_{565} = 0.1) with and without RMF actuation. Data are presented as mean ± s.d. from triplicate measurements.

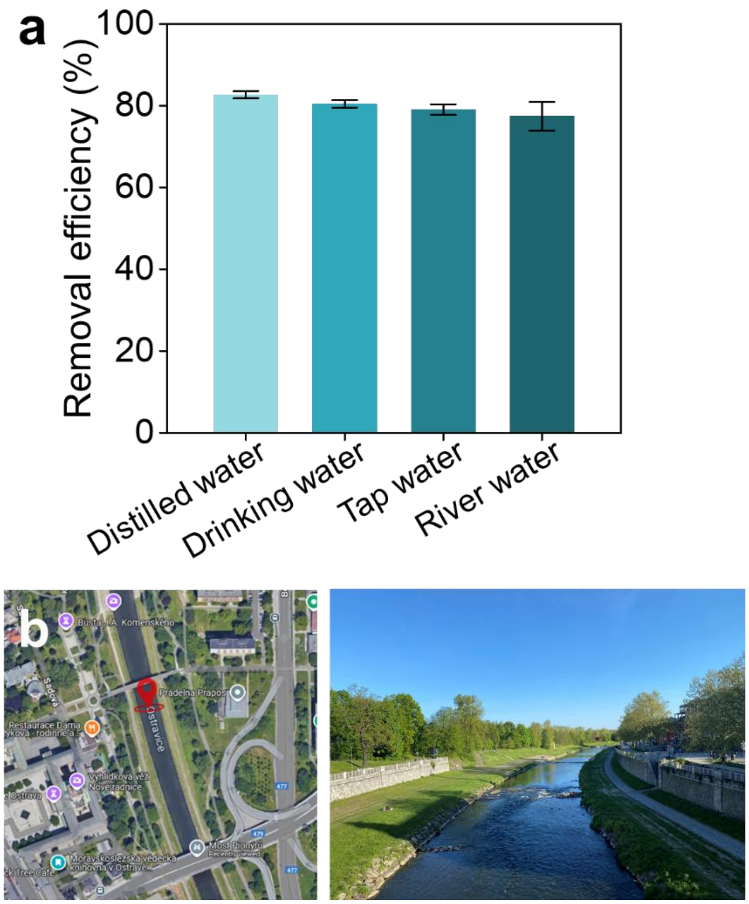

Figure S6. (a) Removal efficiencies of PS microplastics by MTB biobots (OD_{565} = 0.1) actuated under a RMF (5 mT, 0.5 Hz) in various aqueous environments, including distilled water, drinking water, tap water, and river water. (b) Geographical location of the river water sampling site (the Ostravice River, Ostrava, Czech Republic). Data in (a) are presented as mean ± s.d. from triplicate measurements.

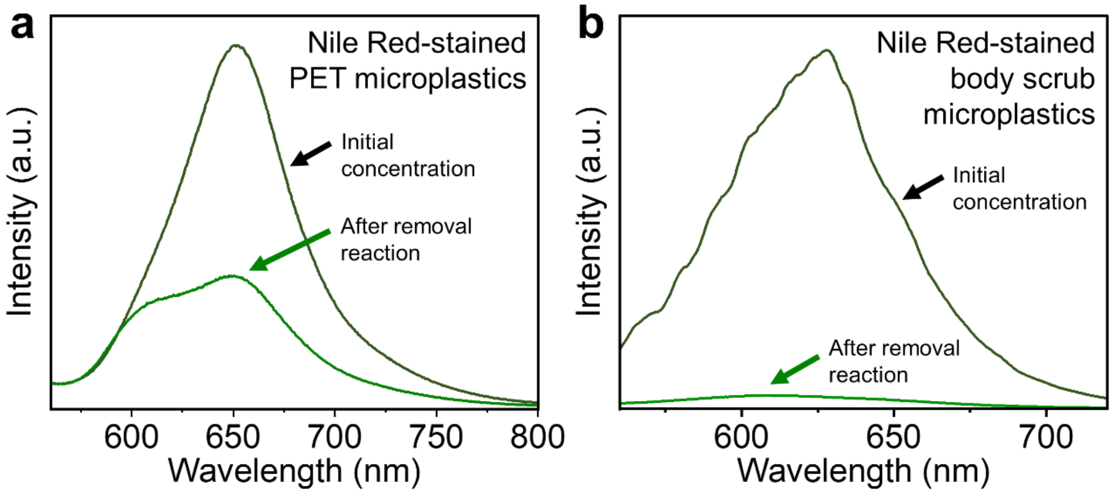

Figure S7. Fluorescence spectra of Nile Red-stained PET microplastics (a) and Nile Red-stained body scrub microplastics (b) before and after the treatment using magnetically driven MTB biobots.

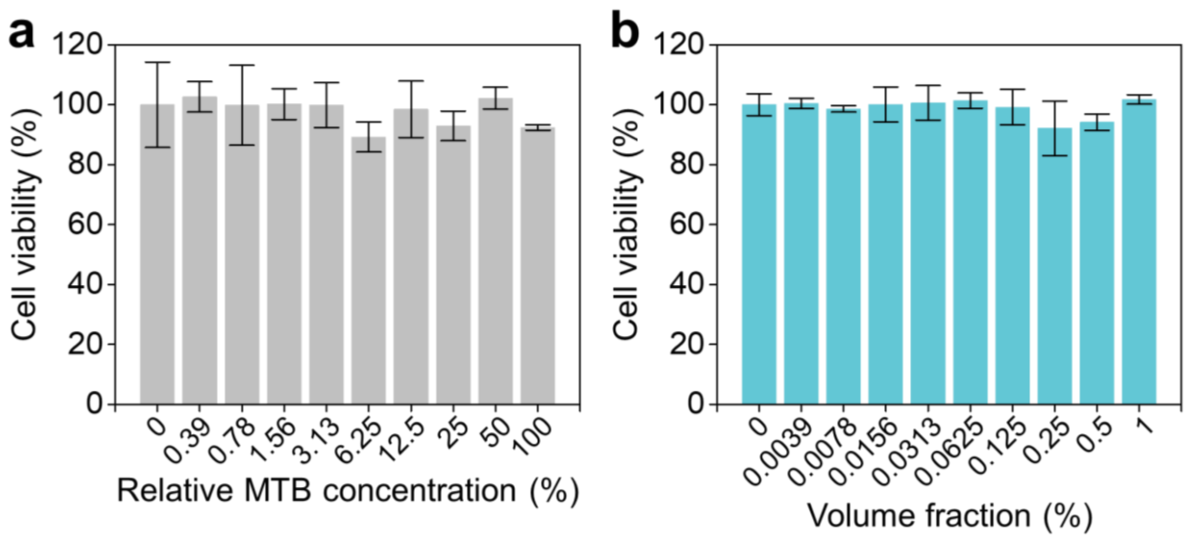

Figure S8. Cytotoxicity evaluation of MTB biobots and supernatant solution after MTB biobot operation. Cell viability of HT1080 cells was evaluated using the MTS assay after 24 hours of exposure to MTB biobots and the supernatant collected after magnetic actuation and retrieval. Data are presented as mean ± s.d. from triplicate measurements.

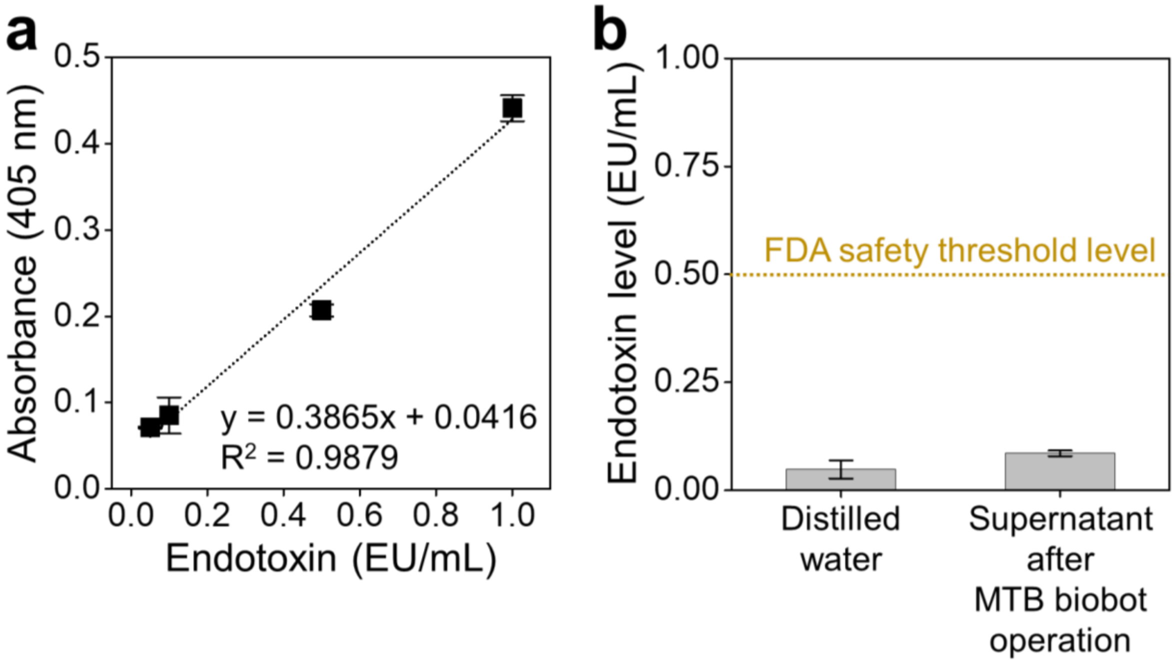

Figure S9. Quantification of endotoxins in the supernatant following MTB biobot operation. (a) Calibration curve based on *E. coli* O111:B4 endotoxin standards (0.05-1.0 EU/mL) used for quantifying endotoxin levels in the test samples. (b) Comparison of endotoxin concentrations in the negative control (distilled water) and the supernatant obtained after MTB biobot operation. The FDA-recommended safety threshold is indicated by the yellow dotted line (0.5 EU/mL). Data in (b) are presented as mean ± s.d. from triplicate measurements.
